# Informal Dementia Caregiver Outcomes in Relation to Care Setting and Loneliness- An Integrative Review

**DOI:** 10.1093/geroni/igaf122.3610

**Published:** 2025-12-31

**Authors:** Maria Weibel, Judith Tate

**Affiliations:** The Ohio State University, Columbus, Ohio, United States; The Ohio State University, Columbus, Ohio, United States

## Abstract

**Background:**

Globally, loneliness is prevalent and expected to continue increasing. Informal dementia caregivers are lonelier than the general population. Loneliness is associated with greater risk for dementia, cardiovascular disease, depression, and premature mortality. Loneliness has also been associated with greater burden and lower quality of life among caregivers. This review investigates the experience of loneliness among dementia caregivers with a focus on the setting in which the caregiving occurred. The dementia caregiving role does not end when the person with dementia (PWD) transitions to a long-term care (LTC) community. Instead, the caregiver’s responsibilities often just shift. Presently, the dementia caregiving experience for caregivers of PWD in LTC is not well studied. Future research is needed to address the dementia caregiver experience of loneliness in both the home/community and LTC settings.

**Methods:**

We conducted a literature review across the PubMed, Embase, and CINAHL databases.

**Results:**

Caregivers of PWD in the home/ community setting experienced loneliness differently than caregivers of PWD in LTC. Some caregivers in the home/ community setting reflected a sense of duty or a cultural norm to provide care that could contribute to loneliness and prolonged caregiving led to increased loneliness. Caregivers whose PWD lived in LTC demonstrated a continuation of the caregiving role and loneliness due to the absence of the PWD.

**Conclusion:**

Identifying the differing risk factors, antecedents, and implications associated with the experience of loneliness by dementia care setting is essential to undertaking a comprehensive approach towards improving the caregiving experience for informal dementia caregivers.

